# Does sadness impair color perception? Flawed evidence and faulty methods

**DOI:** 10.12688/f1000research.9202.1

**Published:** 2016-07-21

**Authors:** Alex O. Holcombe, Nicholas J. L. Brown, Patrick T. Goodbourn, Alexander Etz, Sebastian Geukes

**Affiliations:** 1 School of Psychology, University of Sydney, New South Wales, 2006, Australia; 2 Department of Health Sciences, University Medical Center, University of Groningen, Groningen, 9713 GZ, Netherlands; 3 School of Psychological Sciences, University of Melbourne, Victoria, 3010, Australia; 4 Department of Cognitive Sciences, University of California, Irvine, CA, 92697-5100, USA; 5 Institut für Psychologie, Westfälische Wilhelms-Universität Münster, Münster, 48149, Germany

**Keywords:** Mood, perception, color, open data, reanalysis

## Abstract

In their 2015 paper, Thorstenson, Pazda, and Elliot offered evidence from two experiments that perception of colors on the blue–yellow axis was impaired if the participants had watched a sad movie clip, compared to participants who watched clips designed to induce a happy or neutral mood. Subsequently, these authors retracted their article, citing a mistake in their statistical analyses and a problem with the data in one of their experiments. Here, we discuss a number of other methodological problems with Thorstenson et al.’s experimental design, and also demonstrate that the problems with the data go beyond what these authors reported. We conclude that repeating one of the two experiments, with the minor revisions proposed by Thorstenson et al., will not be sufficient to address the problems with this work.

## Introduction

Based on two experiments, Thorstenson *et al.* (2015a) claimed that a state of sadness—induced by watching a short film clip—impairs performance on a specific perceptual task: discrimination of colors along the blue–yellow axis, but not the red–green axis. This conclusion is interesting because it is specific to a single dimension of color space; poor performance on tasks generally, or low willingness to cooperate with an experimenter, would not be a surprising effect of sadness.

In their retraction notice ( Thorstenson *et al.*, 2015b), the authors acknowledged that their data did not justify their conclusion that impairment was specific to one aspect of color space. They also described an anomaly in the histogram of the data of Experiment 2. In our sections below entitled “A confounded comparison” and “Perceptual impairment or change in bias?”, we detail other problems with the way the experiments were conducted, the choice of stimuli, and the measures chosen. It is these problems that lead us to believe that even the revised Experiment 2 (proposed by Thorstenson *et al.*, 2015b) will not justify their original conclusion. In our “Re-analysis of data” section, we describe a number of statistical issues that go beyond the problem mentioned in the retraction notice. In our final section, “Anomalies and strange patterns in the data”, we report some other anomalies with the dataset, which further undermine confidence in the way the experiments were carried out and in the resulting findings. We offer this analysis not only to improve the state of the literature on the perceptual effects of watching sad film clips, but also in the hope that it will lead to better work in this area in the future.

## A confounded comparison

When designing an experiment comparing two conditions, one strives to make the factor of interest the *only* difference between the conditions. Thorstenson *et al.* (2015a) contrasted two film clips, one of which was intended to cause the participants to feel sad. The clips ought to have been chosen to avoid any other differences (on average) in their effect on the participants. The “sadness” clip of Experiment 1 is an excerpt from the animated Disney movie *The Lion King*, with an unusual lighting that gives the impression of daylight filtered through dust, while the “happiness” clip is a warmly-lit, indoor recording of the comedian Bill Cosby. The “sadness” clip used in Experiment 2 is the same Lion King excerpt, converted from color to grayscale, and the “neutral” clip is a grayscale film of sticks appearing on top of one another at different orientations (also converted from color to grayscale).

Unfortunately, differences in mean color and the color variability in these clips may have differently affected subsequent perception of blue and yellow versus red and green. For example, the contrast along the blue–yellow axis might have been greater in the sadness clips. Such a difference would result in reduced sensitivity to blue–yellow ( Krauskopf *et al.*, 1982). The use of grayscale clips does not eliminate this issue. An analysis of the Hue, Saturation, Brightness (HSB) values in the movie files posted online indicates that the mean color of the grayscale clips is bluish-reddish, with some saturations approaching 5%. These grayscale clips, therefore, may have had differences in average color as well as color contrast that were uncontrolled. To resolve the issue, the colors displayed on the laboratory screen must be measured with a colorimeter. The authors should have made these measurements and reported them in their paper, in order to provide an indication of whether simple contrast adaptation specific to each color axis would occur when viewing the clips. In the absence of a report of these measurements or any mention of the issue, it appears that Thorstenson *et al.* (2015a) did not take the appropriate steps to eliminate the possibility that a classic process in color perception could explain the results.

Blue, yellow, green and red are all defined relative to a white point that, in the human visual system, is quite flexible. Just as one can adjust the white balance of a camera to fit scenes with different illumination, for humans the point considered to be the center of color space changes depending on the palette of colors that confronts us ( Webster & Leonard, 2008). Unfortunately, Thorstenson *et al.* displayed their test stimuli in a manner unsuited to controlling the participant’s white point. Color perception experiments typically use a neutral grey or white background to provide a white-point reference for participants, alongside the test stimulus. Thorstensen *et al.*’s use of full-field color without a simultaneous reference stimulus makes categorization of desaturated patches problematic. In such circumstances, the participants’ white points may be more dependent on the color content of the movie clip they viewed previously, which as mentioned above appears to have been uncontrolled. In addition, the lack of a grey or white reference stimulus may cause participants to be completely unable to judge the stimulus color more often. In such circumstances, responses may be particularly prone to influence by cognitive factors or by priming ( García-Pérez & Alcalá-Quintana, 2013).

In addition to color, there may be other confounding difference between the two types of clips. The clips likely differed in interest, action, and other features. Unfortunately, it is difficult to know whether such features might have affected participants’ color perception. It certainly is possible for such differences to bias the participants’ responses when they are uncertain of the stimulus’s color. Of course, it is almost impossible to avoid featural differences between any two particular clips. Because of this, a good experimental design would utilize a large set of clips, assess the various featural differences between the stimuli, and either match the two groups of clips carefully on their features, or model them as random effects in a mixed-effects model ( Wells & Windschitl, 1993).

## Perceptual impairment or change in bias?

Thorstenson *et al.* (2015a) concluded that sadness “impair[s] color perception on the blue–yellow color axis” (p. 1). But signal detection theory, which was not used, would be necessary to show whether the decrease in accuracy found was indeed due to an impairment in color perception (i.e., a decline in sensitivity along the blue–yellow axis), or whether the judgments of the sadness group were instead biased away from blue and yellow. For decades, studies of perception have used signal detection theory to distinguish between a change in perceptual ability and a change in, say, cognitive bias to press the blue or yellow button rather than the red or green one ( Green & Swets, 1966). Unfortunately, Thorstenson *et al.*’s plan to simply repeat Experiment 2 with minor revisions would not allow for the appropriate analysis. In Experiment 2, participants were tested in only two trials for each stimulus. Much more data would be needed to discern between a decline in the participants’ ability to discriminate the colors from a decline in the participants’ bias toward pressing the blue or yellow button (instead of the red or green). For the four-alternative categorization task used by Thorstenson *et al.*, a multivariate extension of signal-detection theory should be used (such as general-recognition theory, Ashby & Townsend, 1986).

The analyses of Thorstenson *et al.* (2015a), and also the improved analyses that we have suggested above, assume stochastic independence of participants’ accuracies on the red–green stimuli and the blue–yellow stimuli. Unfortunately, however, this assumption may be unjustified. Participants’ accuracy on one axis might affect their guessing strategy on another. In Experiment 1 for example, accuracy was very high on the blue–yellow axis, suggesting that many participants may have had a clear color percept of the blue or yellow stimuli, but were less certain about the red and green stimuli. If so, when an unclear patch came up and they guessed, they may have been unlikely to guess blue or yellow, in an effort to balance their responses across the available options (many participants may have correctly guessed that the stimuli were roughly equally distributed among the four categories). This would artifactually improve performance on the red and green stimuli. Modeling this phenomenon, however, would be difficult. Even if we had access to the raw responses (rather than the summary data provided by Thorstenson *et al.*), it would be difficult to estimate the participants’ guessing strategy. To avoid this problem in a future version of this experiment, we suggest that Thorstenson and colleagues should consider adopting the two-alternative forced choice design ( Fechner, 1860/1966) commonly used in psychophysics.

Thorstenson *et al.* (2015a) are not the only researchers to have used bias-prone measures of perception to support claims that some non-perceptual state can influence perception. Firestone & Scholl (2015) provide many other examples, with useful discussion.

## Reanalysis of data

There are issues with the dataset ( Thorstenson *et al.*, 2015c) that were not described in the retraction ( Thorstenson *et al.*, 2015b) of the article. Some of these issues affect Experiment 1, which Thorstenson *et al.* indicated that they plan to re-publish. We describe and discuss these issues in this section, as well as the next section, “Anomalies and strange patterns in the data”.

The most important empirical claim made by Thorstenson *et al.* (2015a) was that there was a difference in performance between their two measures, namely color perception along the blue–yellow axis and color perception along the red–green axis. However, these authors provided no statistical test of a difference in the effect of the film clip on blue–yellow compared to red–green. This problem was discussed widely on blogs and on PubPeer (2016), and was acknowledged by Thorstenson *et al.* (2015b) in their retraction notice. Thorstenson *et al.* (2015a, p. 4) noted that the possible difference between red–green and blue–yellow color perception, such that “sadness influenced chromatic judgments about colors on the blue–yellow axis, but not those on the red–green axis,” is critical to ruling out “the possibility that sadness simply led to less effort, arousal, attention, or task engagement.” Such a difference implies a statistical interaction between the “emotion condition” and “color axis” factors. However, the authors did not report a statistical test for this interaction in either of their experiments. When we (and the authors of various blogs, such as Areshenkoff, 2015) tested this interaction with the published data, we found (code at: Holcombe *et al.*, 2016) that it was not statistically significant: Experiment 1, *F*(1, 125) = 3.51, *p* = .06; Experiment 2: *F*(1, 128) = 0.40, *p* = .52. In their retraction notice, Thorstenson *et al.* (2015b) reported a *z* test to test the same issue (for unknown reasons, they did not use a conventional statistical interaction, but instead followed a procedure described in Rosenthal & Rosnow, 1991), which also did not yield statistical significance.

An additional potential source of error is that Thorstenson *et al.* (2015a) did not record the color-perception performance of their participants before the film clips were shown. It was apparently considered sufficient to randomize the participants to watch one of two film clips; presumably the reasoning was that this randomization made it unlikely that the two groups differed much in baseline performance. However, even if this assumption were to be confirmed, the two groups would likely differ somewhat at baseline, even if by only a small amount, and such a difference could have an effect on the outcome given the relatively small sample sizes involved ( Saint-Mont, 2015). It would have been useful for these differences to be measured and included in the subsequent analyses, given that Thorstenson *et al.*’s hypothesis was that sadness would “impair” (i.e., reduce, compared to a previous state) participants’ color perception. In addition, using a change score for each participant can increase statistical power by reducing the contribution of variation among participants to the error term.

Finally, we note that Thorstenson *et al.*’s (2015a) experimental design assumes the complete independence of participants’ accuracy on the two sets of stimuli (red–green and blue–yellow). We discuss a possible violation of this assumption in our “Perceptual impairment or change in bias?” section above.

## Anomalies and strange patterns in the data

### Large numbers of participants with identical scores

**Figure 1.**
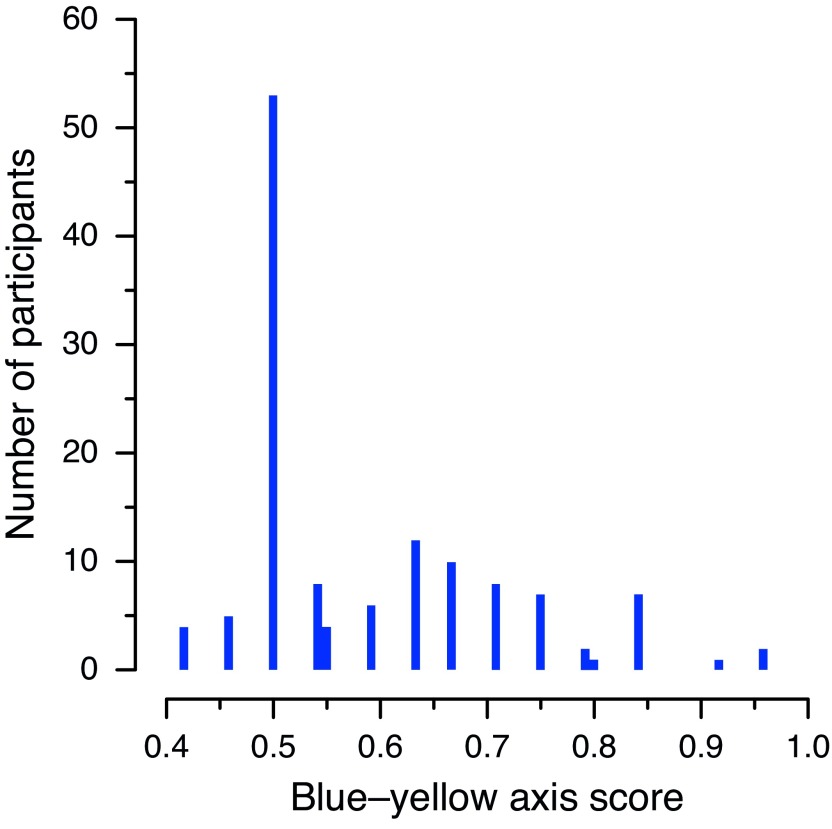
Distribution of standardized blue–yellow axis scores recorded for Experiment 2. The histogram shows the number of occurrences of each score, calculated by Thorstenson *et al.* (2015a) as the proportion of correct responses in the 24 trials in which either a blue or yellow patch was presented. The pairs of adjacent bars near 0.55 and 0.8 correspond to cases that are not compatible with correct rounding.

We observed a strange pattern in the data for the blue–yellow axis in Thorstenson *et al.*’s (2015a) Experiment 2. Specifically, a very large number of participants (53 out of 130) had a score of exactly 50%, corresponding to 12 out of 24 correct responses, with every other number of correct responses (10, 11, 13, 14, etc.) being achieved by a much smaller number of participants. This is illustrated in Figure 1, where the spike at the 50% level is clearly visible. This issue was one of the reasons given by Thorstenson *et al.* (2015b) for retracting their article.

Upon our request, Christopher Thorstenson provided us with *per-color patch data*. The per-color patch data consists of two Excel files (one per experiment), with each cell containing a combined score for the participants’ two responses for each color and saturation level. The score for each case is either 0.0, 0.5, or 1.0, corresponding to 0, 1, or 2 correct responses (see https://osf.io/sbhn9/).

Closer examination of the per-color patch data, shows that of the 53 participants scoring exactly 50%, 49 (i.e., 37.7% of all participants in Experiment 2) had identical scores for both colors, namely 6.0 (100%) for blue and 0.0 (0%) for yellow (in their patch data files, each correct observation counts for a half-point, so that scores for each color range from 0.0 to 6.0 in increments of 0.5; thus, a score of 6.0 corresponds to 12 correct responses out of 12). We are at a loss to explain this phenomenon, which affected both experimental conditions (26 of the 49 participants with this 12–0 split were in the neutral condition, with 23 of the 49 being in the sadness condition). There seems no reason to suppose that the undergraduate participants in this experiment would have been markedly less sensitive to yellow than those in Experiment 1. However, even if their ability to distinguish the color yellow was affected by some environmental factor, or if they had been accidentally (perhaps due to a software problem) shown, say, a gray patch instead of a yellow one, their expected score for yellow would be 1.5 (i.e., three correct identifications out of 12 attempts) by chance alone.

### Inconsistent calculation of percentages

A further concern with the summary data ( Thorstenson *et al.*, 2015c) is that the conversion of color perception values from counts of responses to percentages of correct attempts for both axes in Experiment 2 appears to be inconsistent. These percentage values, reported to two decimal places, ought to be the result of dividing the number of successful attempts on each axis (i.e., the total number of correct identifications of red or green patches for the red–green axis, and the total number of correct identifications of blue or yellow patches for the blue–yellow axis) by 24. For example, an examination of the patch scores shows that participants #4 and #5 both scored a total of 6.5 for blue and yellow patches combined, corresponding to 13 correct identifications out of 24 on the blue–yellow axis. However, in the published dataset file, participant #4 has a value of 0.54 for the corresponding percentage variable BY_ACC (blue–yellow accuracy), whereas participant #5 has a value of 0.55 for the same variable (the true value of 13/24 being $0.5416\bar{6}$). Christopher Thorstenson (personal communication, December 1, 2015) has explained to us that these percentages resulted from taking the mean of the individual percentages of correct attempts for each color of the axis in question (e.g., red and green), with these individual percentages having first been rounded to two decimal places. It is not clear whether this explains all the anomalies in the data for Experiment 2; in any case, it serves as a reminder that, in order to avoid loss of information, rounding should be avoided during an analysis and only applied, if necessary, during the final reporting of results.

### Large differences in skewness between experiments

**Figure 2.**
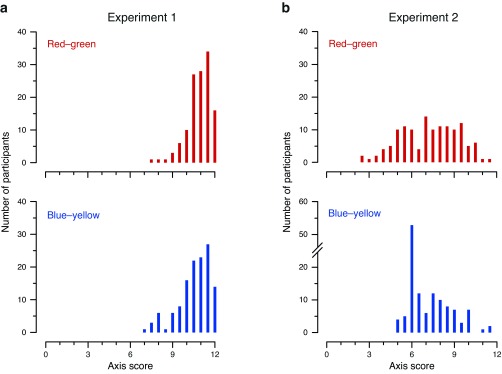
Distribution of blue–yellow and red–green axis scores in ( **a**) Experiment 1 and ( **b**) Experiment 2. Histograms show the number of occurrences of each score for the red–green axis (top panels) and blue–yellow axis (bottom panels). The range of scores on the *x*-axis is 0–12, reflecting Thorstenson *et al.*’s (2015a) scoring scheme of 0.5 points per correct answer, with 12 trials per color and two colors per axis. Note the discontinuity in the *y*-axis for blue–yellow in Experiment 2 (bottom-right panel), added to accommodate the surprisingly high peak at 6.

An examination of the distribution of the scores for the two color axes reveals considerable differences between Experiment 1 and Experiment 2. In Experiment 1, the distribution for both axes was substantially negatively skewed, with the majority of participants correctly identifying almost all of the patches for all four colors ( Figure 2a). In Experiment 2, the score distribution was different for each axis. For the red–green axis ( Figure 2b, top panel) the scores were approximately normally distributed: roughly similar numbers of participants achieved each possible score, with a small number having very low or very high scores. In contrast, the blue–yellow axis was positively skewed, displaying the “spike” discussed previously ( Figure 2b, bottom panel).

**Figure 3.**
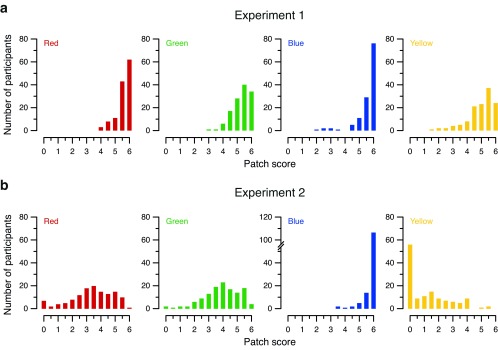
Distribution of color patch scores in ( **a**) Experiment 1 and ( **b**) Experiment 2. Histograms show the number of occurrences of each score for the red, green, blue and yellow color patches. The range of scores on the *x*-axis is 0–6, reflecting Thorstenson *et al.*’s (2015a) scoring scheme of 0.5 points per correct answer, with 12 trials per color. Note the discontinuity in the *y*-axis for blue patches in Experiment 2 (bottom row, second panel from right), added to accommodate the surprisingly high peak at 6.

Using the patch-level data, we broke the two-color axis scores down into individual colors, as shown in Figure 3. For Experiment 1, the per-color data more or less followed the pattern of the two-color axis of which each color was a part ( Figure 3a); this was also true for the red–green axis in Experiment 2 ( Figure 3b, left panels). However, an even stranger pattern emerged for the blue–yellow axis in Experiment 2 ( Figure 3b, right panels). Of the 130 participants, 106 (81.5%) scored a maximum 6.0 (corresponding to 12 correct responses) for blue, while 56 (43.1%) scored zero for yellow. The observed “spike” at 50% (i.e., 12 out of a possible 24 correct responses) for the blue–yellow axis is thus mostly explained by people who had a perfect score (12 out of 12) for blue, while completely failing to recognize yellow patches at any saturation and thus obtaining a score of 0.

**Figure 4.**
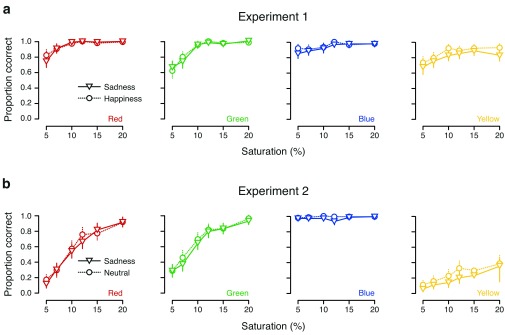
Color categorization as a function of saturation in ( **a**) Experiment 1 and ( **b**) Experiment 2. Each panel shows the proportion of correct responses for each of the six saturation levels for a given color. Mean performance in the *sadness* condition is represented by triangles joined by solid lines, and mean performance in the *happiness* (Experiment 1) and *neutral* (Experiment 2) conditions is represented by circles joined by dashed lines. Error bars show parametric 95% confidence intervals on the means. (Details of the color calibration procedure were not stated by Thorstenson *et al.* (2015a), so it is not clear how to interpret these saturation values.)

Figure 4 plots the participants’ performance for each color, broken down further into the proportion of correct responses for each saturation level (recall that participants were asked to identify colors at each of six different levels of saturation.) In Experiment 1, this resulted in what appears to be a ceiling effect – mean accuracy reaches 90% or more already at the third-lowest color saturation level (.10) and levels off as saturation increased thereafter ( Figure 4a). In Experiment 2, the ceiling effect disappeared for the red–green axis, for which scores on both colors improved approximately linearly with increasing color saturation ( Figure 4b, left panels); however, on the blue–yellow axis, the effect of the split between the two colors is once again clear. The ceiling effect is even more pronounced for blue here than in Experiment 1, while scores for yellow are low even at the highest color saturation level ( Figure 4b, right panels).

It is difficult to imagine what might have caused these results in Experiment 2. The Method section for this experiment suggests that the only change that was made from Experiment 1 was the nature of the film clips that were shown to participants. The differences for both axes (and, indeed, for all four colors) between Experiments 1 and 2—regardless of the film clip watched by participants—are puzzling, given that both samples were drawn from the same population of undergraduates and hence ought not to differ widely in their physiological characteristics. Because the color characteristics of the two sets of film clips were apparently not well-controlled, one possible explanation for this discrepancy is differential adaptation of the color mechanisms in the visual system, which adds to our concern about possible confounds (see our section “A confounded comparison?”). But we have difficulty believing that this, on its own, could account for such a substantial difference between the two experiments.

Given that the extreme blue–yellow scores in Experiment 2 were obtained from participants in both the neutral and sadness conditions, a further possibility is that simply watching grayscale film clips for a few minutes was sufficient to substantially distort participants’ color vision (on the blue–yellow axis only). However, if Thorstenson and colleagues had noticed such a finding, they would presumably have mentioned it in their article (and perhaps alerted colleagues in the field of physiology to this remarkable discovery). Otherwise, we are left with two possible conclusions: either around 40% of the participants in Thorstenson *et al.*’s (2015a) Experiment 2 all had the same problem with their vision (which was not shared by any of the participants in Experiment 1), or some form of equipment failure or other technical problem caused this unusual pattern of values to be recorded. In any case, it seems likely that Thorstenson *et al.* failed to notice this anomaly when examining their data prior to performing their statistical analyses.

## Conclusion

While we strongly support the retraction by Thorstenson *et al.* (2015b) of their article ( Thorstenson *et al.*, 2015a) on the basis of the problems they noted with Experiment 2, we maintain that the basic methodology of both of their experiments is flawed. As Thorstenson and colleagues move forward, together with others who seek to assess whether mood and other factors can influence perception, they should bring their work up to modern standards of statistics and psychophysics. Doing so for experiments like those of Thorstenson *et al.* would involve: (1) careful control of the visual differences between the movie clips, or, better, mood induction via non-visual stimuli such as an audio recording of a story; (2) the use of many movie clips or recordings, and mixed-effects analysis to address differences that cannot be eliminated between any two clips or recordings; (3) a baseline measurement of color perception; (4) an analysis based on signal-detection theory.

## Data availability

Open Science Framework: Reanalysis of Thorstenson *et al.*’s (2015) “Sadness Impairs Color Perception”, doi 10.17605/osf.io/kwuq4 ( Brown *et al.*, 2016).

We have archived the R code that we used to analyze the data and generate our figures at the Open Science Framework (OSF; doi: 10.17605/osf.io/kwuq4). This code works with the original dataset files uploaded to OSF ( Thorstenson *et al.*, 2015c), together with the patch data files that Christopher Thorstenson sent us (by “patch data”, we mean data broken down to the individual color patches tested) and that we posted at https://osf.io/sbhn9/ (Thorstenson subsequently asked us to delete two of the files, which we did).
