# Supplementary material for: Does sadness impair color perception? Flawed evidence and faulty methods
Source: F1000Res. 2016 Jul 21;5:1778. [Version 1] doi: 10.12688/f1000research.9202.1 (PMC4979646; doi:10.12688/f1000research.9202.1)
Supplement: Peer review history

# Peer review: Referee response for version 1

**DOI:** 10.5256/f1000research.9905.r15140

**Published:** 2016-08-08

**Authors:** Michael A Crognale

**Affiliations:** 1 Department of Psychology, University of Nevada, Reno, NV, USA

The manuscript by Holcombe *et al. *as a comment on Thorstenson *et al.'*s prior manuscript is well well written and reasoned.  The problems with the Thorstenson *et al*. article are many and most of them have been well addressed here. However, since the manuscript by Throstenson *et al.* has been retracted reportedly for other reasons, it does not seem particularly fruitful to critique the original manuscript further unless a resubmission has published.  On the other hand, pointing out the additional methodological and statistical problems with the original manuscript may be instructive for those following up on this work as suggested by Holcomb *et al. *In particular, there does seem to be a disturbing trend to attribute results to factors that have not been well established as uniquely or even likely causal, (e.g. in the present case that "sadness" caused the observed trends in the data). Holcomb *et al.'*s manuscript also illustrates the importance of scrutinizing the raw data for evidence of faulty methodology, and ceiling/floor effects. Perhaps publishing the present manuscript may be worthwhile as it is a potentially valuable instructional tool.

I have read this submission. I believe that I have an appropriate level of expertise to confirm that it is of an acceptable scientific standard.

# Peer review: Referee response for version 1

**DOI:** 10.5256/f1000research.9905.r15370

**Published:** 2016-08-02

**Authors:** Sophie von Stumm

**Affiliations:** 1 Department of Psychology, Goldsmiths University of London, London, UK

The article reviews a previous publication that was based on two flawed experiments and has since been retracted. The authors thoroughly analyse the experimental methods that were employed in the retracted study, and they support the retraction. In addition, they highlight further problems with the experimental design and they make specific recommendations for improving future studies in this area.

I have read this submission. I believe that I have an appropriate level of expertise to confirm that it is of an acceptable scientific standard.
